# Synthetic PreImplantation Factor (PIF) prevents fetal loss by modulating LPS induced inflammatory response

**DOI:** 10.1371/journal.pone.0180642

**Published:** 2017-07-12

**Authors:** Nicoletta Di Simone, Fiorella Di Nicuolo, Riccardo Marana, Roberta Castellani, Francesco Ria, Manuela Veglia, Giovanni Scambia, Daniel Surbek, Eytan Barnea, Martin Mueller

**Affiliations:** 1 Department of Obstetrics and Gynecology, Università Cattolica Del Sacro Cuore, A. Gemelli Universitary Hospital, Rome, Italy; 2 International Scientific Institute Paolo VI, ISI, Università Cattolica Del Sacro Cuore, A. Gemelli Universitary Hospital, Rome, Italy; 3 Institute of General Pathology, Università Cattolica Del Sacro Cuore, Rome, Italy; 4 Department of Obstetrics and Gynecology, University Hospital Bern, Bern, Switzerland; 5 The Society for the Investigation of Early Pregnancy (SIEP), Cherry Hill, New Jersey, United States of America; 6 BioIncept LLC, Cherry Hill, New Jersey, United States of America; 7 Department of Obstetrics, Gynecology, and Reproductive Sciences, Yale University School of Medicine, New Haven, Connecticut, United States of America; Xavier Bichat Medical School, INSERM-CNRS - Université Paris Diderot, FRANCE

## Abstract

Maternal control of inflammation is essential during pregnancy and an exaggerated response is one of the underlying causes of fetal loss. Inflammatory response is mediated by multiple factors and Toll-like receptors (TLRs) are central. Activation of TLRs results in NALP-3 mediated assembly of apoptosis-associated speck-like protein containing a CARD (ASC) and caspase-1 into the inflammasome and production of pro-inflammatory cytokines IL-1β and IL-18. Given that preventing measures are lacking, we investigated PreImplantation Factor (PIF) as therapeutic option as PIF modulates Inflammation in pregnancy. Additionally, synthetic PIF (PIF analog) protects against multiple immune disorders. We used a LPS induced murine model of fetal loss and synthetic PIF reduced this fetal loss and increased the embryo weight significantly. We detected increased PIF expression in the placentae after LPS insult. The LPS induced serum and placenta cytokines were abolished by synthetic PIF treatment and importantly synthetic PIF modulated key members of inflammasome complex NALP-3, ASC, and caspase-1 as well. In conclusion our results indicate that synthetic PIF protects against LPS induced fetal loss, likely through modulation of inflammatory response especially the inflammasome complex. Given that synthetic PIF is currently tested in autoimmune diseases of non-pregnant subjects (clinicaltrials.gov, NCT02239562), therapeutic approach during pregnancy can be envisioned.

## Introduction

During pregnancy, allogeneic fetal cells invade the maternal decidua and are protected from the maternal immune system. Controlling the maternal response to inflammation is essential as an exaggerated response is one of the underlying causes of early and even later fetal loss [1]. Fetal loss is associated with multiple causes such as anatomic, genetic, and hematologic disorders but immune defects emerged as central players recently. Not surprisingly, proper immune adaptations play a key role in prevention of pregnancy disorders including preeclampsia, fetal growth restriction, and premature birth [2].

One of the essential players of an immune system during pregnancy is a set of pathogen recognition receptors: toll-like receptors (TLRs) in a trophoblast. Once TLRs are activated by pathogen associated molecular patterns (PAMPs), robust activation of NFκ-B and MAP kinase signaling pathways induce up-regulation of associated genes. These genes are mostly pro-inflammatory and NALP–inflammasome is central [3, 4]. To date four main types of inflammasomes have been described including the NALP-3 subtype. Upon activation of NALP-3, apoptosis-associated speck-like protein containing a CARD (ASC) and caspase-1 are assembled into the inflammasome [4, 5]. This multi-protein complex enables the caspase-1-mediated proteolytic processing of the pro-inflammatory cytokines IL-1β, IL-18 and IL-33, thus generating their respective mature secreted forms. All of these events are necessary during inflammatory response [5].

The lipopolysaccharide (LPS) induced inflammation is a well-documented and frequently used model to study induced fetal loss [6, 7]. The bacterial antigen LPS is a PAMP in the extracellular milieu. It induces macrophage-derived TNF-α production which in turn activates NK cells and IFN-γ secretion resulting in a positive feed-back [8]. This pathway leads to activation of the uterine and placental endothelium and the release of embryotoxic cytokines [9, 10]. We hypothesize that counteracting this adverse environment through targeted therapy would constitute important progress in pregnancy management and fetal loss prevention. A potent candidate in fetal loss prevention is PreImplantation Factor (PIF) as PIF regulates the inflammatory response during pregnancy [11]. PIF is a 15-amino acid peptide secreted by the embryo and is associated with favorable pregnancy outcome [12–15]. PIF`s essential role during pregnancy is supported by the ability to promote embryo development, endometrial receptivity, and trophoblast invasion [16–21]. Interestingly, PIF interaction with maternal immune system goes beyond binding to CD14^+^ T regulatory cells as it targets the adaptive arm of immunity (CD3^+^ cells) as well [22]. PIF`s mode of action is mediated in part by reducing oxidative stress and protein misfolding, thereby protecting against embryo toxicity [20, 21]. In addition, PIF reduces natural killer cells toxicity by down-regulating the pro-inflammatory CD69 expression [23]. Together, PIF is a unique and pregnancy essential peptide which possesses immune-modulatory and not immune-suppressive properties. Not surprisingly, synthetic PIF (PIF analog) protects against multiple immune disorders [24–30] and received a FAST-Track FDA approval for clinical trial in autoimmune diseases of non-pregnant subjects (clinicaltrials.gov, NCT02239562).

Whether synthetic PIF administration could be effective in improving pregnancy-induced pathologies *in vivo* is not yet established. In view of its translational potential, we used an intact immune murine model to study the mechanisms of LPS-induced fetal loss and examined synthetic PIF’s potential as a treatment.

## Materials and methods

Synthetic PIF_15_ (MVRIKPGSANKPSDD) was synthesized by solid-phase peptide synthesis (Peptide Synthesizer, Applied Biosystems) employing Fmoc (9-fluorenylmethoxycarbonyl) chemistry at Bio-Synthesis, Inc. (Lewisville, TX, USA). Final purification was carried out by reversed-phase HPLC and identity was verified by matrix-assisted laser desorption/ionization time-of-flight mass spectrometry and amino acid analysis at >95% purity. Anti-PIF monoclonal antibody against MVRIKPGSANKPSDD was generated in (Genway, SanDiego, CA, USA).

### Animals and treatments

Female Swiss mice (7–8 weeks old) were supplied from Centro Ricerche Sperimentali (CENRIS), Università Cattolica del S. Cuore, Roma and were paired with adult Swiss mice; the day of appearance of post-coitum vaginal plug was considered as day 0 of gestation. Animals were housed in accordance with Ethics Committee and Veterinary Department guidelines. Acclimatization of animals to the laboratory environment was allowed prior to surgery. Aseptic rodent survival surgery guidelines were followed. Animals received food and water ad libitum and were housed under controlled conditions of light (12h light/12h dark) and temperature (23–25°C). In preliminary experiments we tested several doses of LPS (from 0.01 μg/g to 1.0 μg/g; from Escherichia Coli serotype 0111:B4; Sigma-Aldrich, St Louis MA, USA) to delineate its ability to induce fetal demise. In our model, we choose the LPS concentration that was able to induce ~ 80% of fetal loss (0.1μg/g).

**Fig 1:**
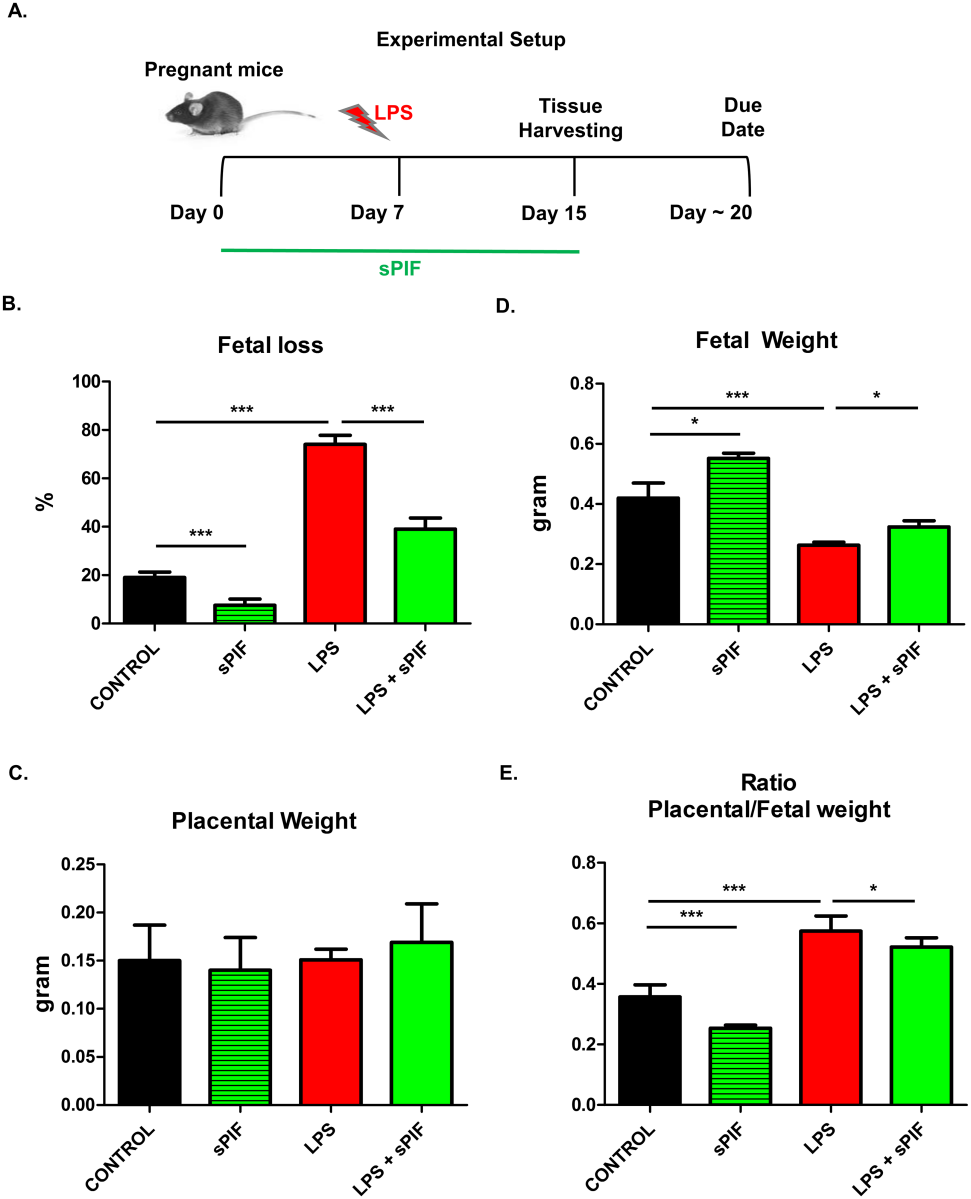
Experimental setup and fetal outcomes after LPS induced insult and synthetic PIF treatment. **(A)** Experimental setup: We used 4 experimental groups (n = 18 pregnant animals each group). Control group received PBS (200ul/day from postnatal day 0 until day 15 (P0-15) and 200ul PBS on P7. Synthetic PIF group received synthetic PIF (1ug/g mouse/day) from P0-15 and PBS on P7. LPS group received PBS from P0-15 and LPS (0.1ug/g mouse) on P7. LPS+sPIF group received synthetic PIF and LPS (as above). Fetal outcomes are presented in (**B)** fetal loss, (**C)** placental weight, and **(D)** fetal weight, and (**E)** Ratio placental/fetal weight. *p<0.05, **p<0.01, and ***p<0.001. sPIF: synthetic PreImplantation Factor; LPS: Lipopolysaccharides. Data are mean ± SD.

The experimental protocol included 4 groups (n = 18 pregnant animals each group; Fig 1A). Two groups of pregnant mice were treated with synthetic PIF (1μg/g mouse /day) or with phosphate buffered solution, PBS (200 μl, control group) using micro-osmotic pumps from day 0 until day 15 of gestation. Briefly, mice were anesthetized via intraperitoneal injection of Ketamine and Xylazine (ketamine 80–100 mg/kg, xylazine 10–12.5 mg/kg). Once the animal has lost its righting reflex we proceed with surgical preparation of implantation site, pumps were implanted subcutaneously on the back of mice by making a small cut in the mid-scapulary region and incision was closed with wound clip. After recovery from anesthesia, mice were monitored for several signs including bleeding, discomfort, or pain. If needed, to alleviate postoperative pain, local anesthesia was used successfully (lidocaine, 4 mg/kg, 0.4 mL/kg of a 1% solution). Notably, the dosage of synthetic PIF was used in multiple animal studies previously [24, 25, 27, 28, 30]. Additionally, on day 7 of gestation, each of these two groups was injected, intra-peritoneum, with LPS (0.1 μg/g mouse/200 μl PBS) or PBS (200 μl). Thus, following 4 groups were investigated (Control, synthetic PIF, LPS, and LPS+synthetic PIF). All mice were sacrificed on day 15 of pregnancy, the uteri were dissected and placentae were harvested. Briefly, mice were anesthetized via intraperitoneal injection of Ketamine and Xylazine (ketamine 80–100 mg/kg, xylazine 10–12.5 mg/kg) and sacrificed by cervical dislocation. The number of viable and resorbed embryos was recorded. The fetal loss rate was calculated as follows: Loss rate = (number of demised fetuses/number of total fetuses) x 100. The weight of viable fetuses and placentae were recorded and placental/fetal weight ratio calculated. The placentae were further used for western blot, immunohistochemistry, and cytokine analysis. The blood samples were centrifuged and the serum was stored at -80°C.

All procedures followed the requirements of Commission Directive 86/609/EEC concerning the protection of animals used for experimental and other scientific purposes. All the experimental procedures were approved by the local ethical committee on preclinical studies [n° 5647/14 (A13 D)] Universita`Cattolica del Sacro Cuore Roma, Italy.

### Placental PIF immunofluorescence

Endogenous PIF was detected in placental tissue using immunofluorescence. Briefly, placental samples were fixed in formalin and processed through embedded paraffin for the histological evaluation. Paraffin sections (3 μm) were dewaxed in Histosol (Sigma Chemical Co; St Louis, MO) and rehydrated through descending grade of alcohol (95–70%) to distilled water (dH2O). PIF was detected with a Biotin conjugated antibody against PIF, (Biosynthesis Code: AB1473-1) dil. 1:100 and a secondary reagent Alexa Fluor^®^ 633 streptavidin (biotin-binding protein) (Thermo Fisher Scientific Code: S-21375, Rockford IL. USA) dil. 1: 200 and counterstained using hematoxylin and eosin (H&E). Finally, samples were mounted with EMS Shield Mount (Electron Microscopy Sciences Code: 17985–150). All images were obtained with a DM6000 B microscope (Leica Microsystems) at 20x magnification in a blinded fashion.

### SDS–PAGE and immunoblotting

Placental tissues were collected (approximately 13 from each pregnant mouse), washed with PBS, minced, and lysed using 1% NP40 in the presence of protease inhibitors (Roche Diagnostics, Indianapolis, IN, USA). Protein concentrations were calculated by the BCA assay (Pierce Biotechnology, Rockford, IL, USA). For Western blotting 18–20 placentae were taken randomly from each group and 50 μg of total lysates were separated by 10% SDS-PAGE electrophoresis under reducing conditions. After gel electrophoresis and transfer of proteins to a nitrocellulose membrane, nitrocellulose sheets were blocked at room temperature for 1 h in 5% non-fat dry milk, and incubated overnight at +4°C with a specific primary antibody (anti-NALP-3, or anti- apoptosis-associated speck-like protein containing a CARD (ASC), 1:200, ThermoFisher Scientific, Rockford, IL, USA). The membranes were washed with PBST and incubated in specific horseradish peroxidase-conjugated IgG diluted 1:2000 in 5% non-fat dried milk in PBST. Bound secondary antibody was detected by chemiluminescence. Bands were analyzed with the use of a Gel Doc 200 Image Analysis System and quantified with the use of Quantity One Quantitation Software (both from BioRad). The level of NALP-3 or ASC was estimated versus the constant level of a 42-kDa protein present in the cytosolic extract (β-actin), which was identified with the use of a mouse monoclonal anti-human β-actin antibody (Sigma-Aldrich, St Louis, MO, USA).

### ELISA assay

Caspase-1 levels were measured in lysates obtained from placentae (18–20 placentae taken randomly from each group) by an enzyme-linked immunoassay (ELISA) according to manufacturer’s instructions (USCN Life Science Inc. and Cloud-Clone Corp. Houston, TX, USA). Briefly, samples or standard (100 μl) were added to each well coated with monoclonal anti-caspase-1 antibody. After 2h of incubation at 37°C, wells were washed and incubated with a specific enzyme-linked polyclonal antibody, horseradish peroxidase. Then, tetramethyl-benzidine substrate solution was added to each well, and the color developed in proportion to the amount of the proteins bound in the initial step. The plate was read on a Titertek Multiscan plus Mk II plate reader (ICN Flow Laboratories, Irvine, CA) measuring the absorbance at wavelengths of 450 nm.

### Multiplex bead array analysis

Placental tissues (18–20 placentae taken randomly from each group) were washed with PBS, minced and lysed using 1% NP40 in the presence of protease inhibitors (Roche Diagnostics, Indianapolis, IN, USA). The placental extract supernatant and serum inflammatory cytokines and chemokines (TNF-α, IFN-γ, IL-1b, IL-18, GM-CSF, GRO, Eotaxin, IL-2, IL-4, IL-5, IL-6, IL-9, IL-10, IL12p70, IL-13, IL17a, IL-22, Il-23, IL27, IP10, MCP1, MCP3, MIP1α, MIP2, MIP1β, RANTES) were analyzed using a Multiplex Bead Array System, Procarta^®^ Immunoassay Kit (eBioscience). Briefly, 50 μl of sample or standard solution were added to 25 μl of the bead mixture in a well on the plate. Serum or placental lysates samples or recombinant standard were then allowed to bind to their respective primary antibodies on the spheres during the 2 hours of incubation. After washing, a mixture of biotinylated secondary antibodies was added to each well and allowed to bind to the captured analyte on the beads. After removal of excess antibodies, streptavidin-RPE (fluorochrome) was added and allowed to bind to the biotin on the secondary antibodies during the third incubation step. The excess streptavidin-RPE was washed away and the fluorescence of the bead (which identifies the immunological marker) and the RPE fluorescence were quantitated. The RPE fluorescence was directly proportional to the concentration of each analyte present in the original sample. The analyses of levels of cytokines were made using the Luminex 100 instrument (Luminex Corp., Austin, TX, USA) and STarStation software (V1.1, Applied Cytometry Systems, Sheffield, UK).

### Statistical analysis

The results are presented as the mean ± standard deviation (SD). The data were analyzed using one-way analysis of variance (ANOVA) followed by a post–hoc test (Bonferroni test). Statistical significance was determined at p<0.05.

## Results

### Synthetic PIF prevents fetal loss

Given the endogenous PIF pro-pregnancy properties *in vitro* [14], we examined whether synthetic PIF can replicate its functions *in vivo* as well. We used a well-established model of LPS induced fetal loss (Fig 1A) [6, 7]. We decided to use this model because of the known optimal reproductive outcome in these mice. Therefore, changes in fetal survival can be related to synthetic PIF treatment. We administered LPS on day 7 of gestation and analyzed pregnancy outcome on day 15 of gestation (expected time of delivery day 18–22) mimicking early inflammatory insult during pregnancy (Fig 1A) [7, 31]. We detected an increased fetal loss rate in mice treated with LPS compared with mice injected with PBS alone (Fig 1B compare red to black bars) and synthetic PIF treatment reduced the LPS induced fetal loss significantly (Fig 1B compare green to red bars). Interestingly, PIF reduced fetal loss as compared to control mice as well (Fig 1B compare green to black bars). We concluded that in an inflammatory compromised pregnancy synthetic PIF treatment results in increased fetal survival and decided to evaluate fetal changes next.

To determine the PIF`s effect on the fetuses, we evaluated placental and fetal weights next. Although we did not detect significant differences in placental weight (Fig 1C), synthetic PIF treatment increased the embryo weight compared to LPS treated or control animals (Fig 1D compare green to black and red bars). In further analysis, we determined the ratio of placental to fetal weight in each group. We detected an increased ratio after LPS insult (Fig 1E compare red to black bars) and decreased after synthetic PIF treatment (Fig 1E compare green to black and red bars). This observation is quite intriguing as it suggests that although the placental weight did not change the LPS induced fetal loss was due to placental alterations [32]. To further understand PIF’s effect on the LPS induced insult in the placenta, we tested PIF expression next.

### LPS leads to PIF expression in the placenta

**Fig 2:**
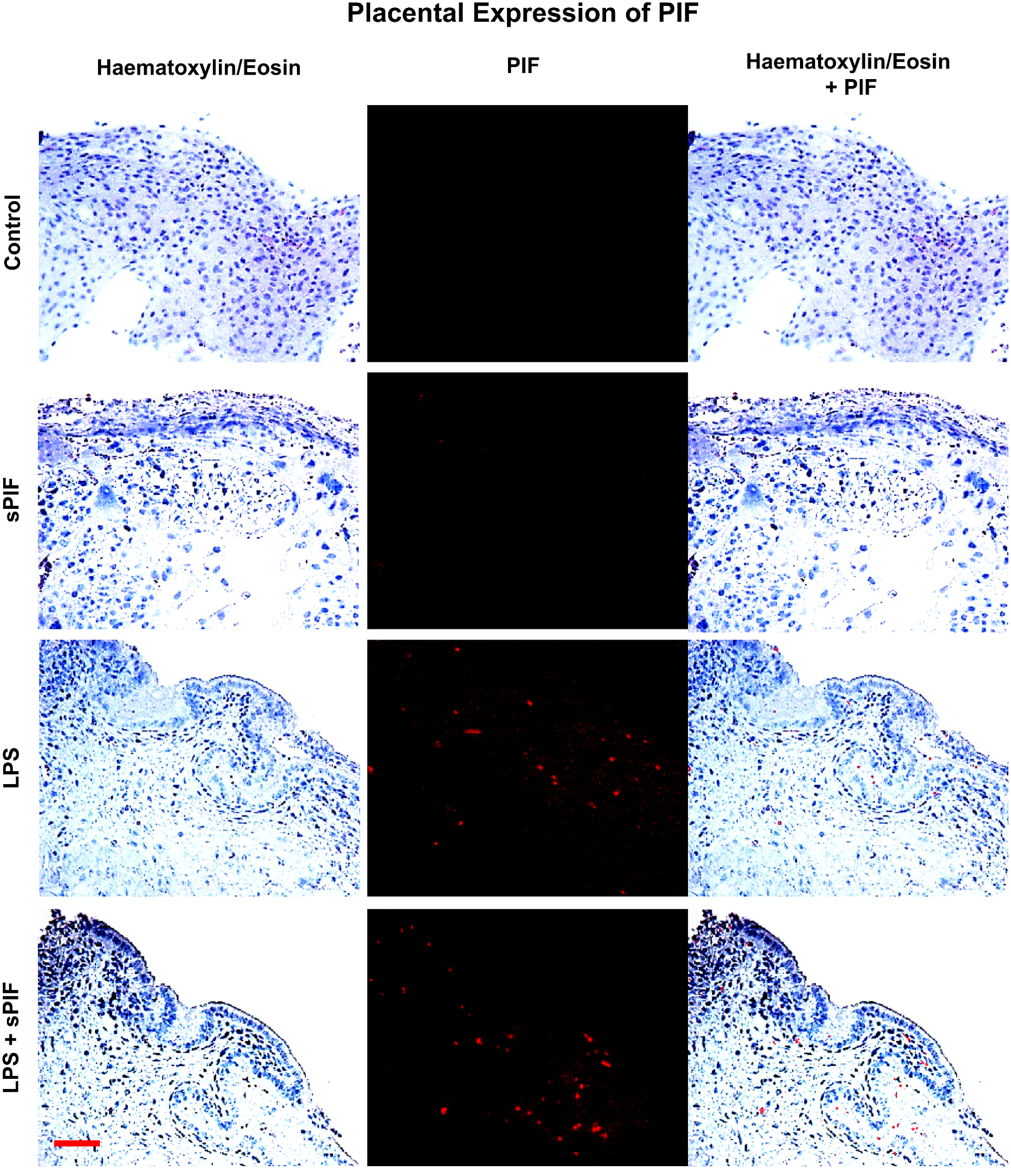
Placental PIF expression. Images of representative placental section (we evaluated 3 consecutive placental sections from 8–10 mice per group) stained using hematoxylin and eosin **(left panels)** and examined the presence of PIF by specific anti-PIF antibody (red immunofluorescence: **middle panels**). Merged images are the **right panels**. We detected no PIF positive cells in Control and synthetic PIF groups **(two upper panels)**. LPS induced inflammatory insult during pregnancy resulted in PIF expression and additional synthetic PIF treatment did not further increase this expression **(two lower panels)**. sPIF: synthetic PreImplantation Factor; LPS: Lipopolysaccharides. Scale bar: 50 μm.

We examined the presence of PIF positive cells by specific anti-PIF monoclonal antibody at day 15 of gestation in the placentae. In line with previous studies [19], we detected minimal to no PIF expression in synthetic PIF or control mice in late gestation (Fig 2: compare two upper panels). Surprisingly, LPS treatment resulted in increased endogenous PIF expression in the placenta and this expression was not additionally amplified by synthetic PIF administration (Fig 2: compare two lower panels). Notably, PIF expression was noticed predominantly in the cytotrophoblast compartment which is in line with previous reports [15]. We postulate that increased endogenous PIF expression may reflect a protective response to the LPS induced inflammatory insult. Further experiments will address the LPS-induced changes in the placenta during embryo development but are beyond the scope of the manuscript. As this endogenous PIF expression did not result in prevention of fetal loss (Fig 1B) and cytokines play a critical role in prevention of fetal loss, we focused on local (placenta) and global (serum) cytokine/chemokine levels next.

### PIF attenuates LPS induced inflammatory signature and inflammasome activation

Given that cytokines play a critical role in the inflammatory response and the placenta is a well-defined source of cytokine production, we tested the placental cytokine/chemokine profile first. We used a multiplex bead array assay to detect levels of 22 cytokines/chemokines. As shown in Table 1 (placental cytokines), LPS administration resulted in increased levels of Tumor necrosis factor (TNF)-α (prime pro-inflammatory cytokine), IL-18 (an inflammasome-related cytokine), and growth related oncogene (GRO: neutrophil-attractive chemokine) and synthetic PIF prevented these increase significantly. To further test the hypothesis of divergent local (placenta) and systemic (serum) inflammatory control, we tested serum cytokine profile as well (Table 2). Indeed, the global inflammatory response was much stronger than in the placenta (Table 2: 14/22 circulating cytokines increased after LPS) and synthetic PIF restored 11 of the 14 LPS induced cytokines/chemokines (Table 2: IFN-γ, IL-18, GM-CSF, GRO, IL-4, IL-5, IL-12p70, IL-17a, IL-22, IL-27, and MIP-1β). Notably, we detected changes after synthetic PIF administration independent of LPS insult as well. For example synthetic PIF up-regulated eotaxin, a chemokine which is involved in placental implantation process [33].

**Table 1:** Placental inflammatory signature.

Cytokine	Control	sPIF	LPS	LPS+sPIF
**TNFα**	9.6±1	8.2±0.9	14.4±0.8*	12.1±1*§
**IFNγ**	1.2±1.5	1.42±1.2	1.8±1.5	1.7±1.5
**IL-1β**	4.7±0.5	2.8±0.4*	5.2±0.3	4.5±1.2
**IL-18**	203±23.2	190±21.6	335±20.4*	**223±24.4**§
**GM-CSF**	ND	ND	ND	ND
**GRO**	1060±159	1255±218*	1686±401*	**1335±550**§
**EOTAXIN**	12.5±1.4	20.2±1.2	11.6±6	26.6±19.8
**IL-2**	ND	ND	ND	ND
**IL-4**	ND	ND	ND	ND
**IL5**	2.3±0.73	4.41±0.5*	5.2±1.2*	4.05±0.9
**IL-6**	ND	ND	ND	ND
**IL-9**	ND	ND	ND	ND
**IL10**	13.9±1.9	5.9±5.4	12.08±7.2	22.7±17.9
**IL12p70**	0.13±0.06	0.82±0.7*	0.35±0.54	0.2±0.12
**IL-13**	ND	ND	ND	ND
**IL-17a**	0.67±0.5	0.83±0.4	0.75±0.5	0.92±0.7
**IL-22**	ND	ND	ND	ND
**IL-23**	279.7±58.8	96.7±28.3*	208.7±48.7	204.7±61.8
**IL-27**	20.7±2.4	17.7±3.4	18.5±1.5	17.3±3.2
**IP10**	14.3±1.5	16.3±3.5	13.1±7	14.4±6.3
**MCP1**	97.4±88	214.1±34	139.3±78	105±86
**MCP3**	173.4±6.7	191±19	152±10	186±16
**MIP1α**	7.6±0.6	8.5±4.4	8.1±3.01	8.5±2.7
**MIP2**	13.6±1.16	14.6±2.3	15.8±1.36	17.7±1.53
**MIP1β**	3.3±1.3	4.9±0.7	3.5±2.4	4.6±0.8
**RANTES**	11.7±1.6	11.4±10.1	13.8±10.2	12.6±39.1

Cytokine levels were measured in placental lysates obtained from mice treated with PBS (Control), synthetic PreImplantation Factor (sPIF), Lipopolysaccharides (LPS), and LPS+sPIF by multiplex bead array assay. The results are expressed as ng/ml (Mean ± SD). Statistical significance:

* P<0.05 vs CTR;

^§^ P<0.05 vs LPS.

Significant changes are marked in bold.

**Table 2:** Serum inflammatory signature.

Cytokine	Control	sPIF	LPS	LPS+sPIF
**TNFα**	6.6±0.4	5.6±1.1	8.6±0.9*	7.7±1.0
**IFNγ**	3.16±0.2	3.39±1.4	16.6±1.6*	**5.92±1.1**§
**IL-1β**	3.4±1	2.5±1.1	5.2± 1.3*	4±1.3
**IL-18**	266±65	150±25*	369±26*	**268±64**§
**GM-CSF**	6.4±1	5.3±0.7	11.06±1.6*	**8.2±1.5**§
**GRO**	15.26±1.8	18.06±2.2	24.9±2.9*	**17.07±1.2**§
**EOTAXIN**	278±61	486±46*	226±37	**430±20**§
**IL-2**	ND	ND	ND	ND
**IL-4**	2.53±0.48	1.74±0.9	7.72±0.2*	3.95±0.3*§
**IL-5**	3.98±0.5	2.66±0.4*	13.7±0.11*	6.8±0.5*§
**IL-6**	ND	ND	ND	ND
**IL-9**	ND	ND	ND	ND
**IL-10**	7.68±1.1	6.65±1.1	11.69±2.2	8.23±1.3
**IL12p70**	1.36±0.49	0.94±0.7*	3.14±1.73*	2.07±1.2*§
**IL-13**	ND	ND	ND	ND
**IL-17a**	5.52±0.06	4±0.7	13.6±0.54*	10.4±0.12*
**IL-22**	35.89±2.1	15.9±27*	47.3±5.6*	**36.2±2.6**§
**IL-23**	36.29±8.6	21.6±2.3*	66.9±3.2*	50.9±5.2
**IL-27**	22.7±1.1	13.4±1.08*	54.5±2.7*	**38.7±3.1**§
**IP10**	28.6±11.2	41.5±12.8	22.5±12.7	35.8±14.6
**MCP1**	47.04±3.3	34.1±2.7*	49.01±1.9	41.8±3.9
**MCP3**	176.8±71	146.4±35	168.4±58	167.3±93.2
**MIP1α**	2.6±1	3.08±0.62	3.1±0.96	3.3±1
**MIP2**	24.7±2.9	24.9±3.4	27.6±4.1	26.3±1.5
**MIP1β**	3.95±0.3	1.9±0.7*	6.4±0.4*	**3.6±0.8**§
**RANTES**	14.2±8.4	22.2±8.5	21.6±7.7	26.5±11.7

Cytokine levels were measured in serum obtained from mice treated with PBS (Control), synthetic PreImplantation Factor (sPIF), Lipopolysaccharides (LPS), and LPS+sPIF by multiplex bead array assay. The results are expressed as ng/ml (Mean ± SD). Statistical significance:

* P<0.05 vs CTR;

^§^ P<0.05 vs LPS.

Significant changes are marked in bold.

We hypothesize that increased endogenous PIF expression in the placenta (Fig 2) results in the divergent inflammatory response at placental and peripheral levels (Tables 1 and 2). However, as in case of progesterone supplementation [34], additional synthetic PIF supplementation is necessary to prevent both the local and peripheral LPS-induced response (Tables 1 and 2) and therefore provide a protective effect (Fig 1).

**Fig 3:**
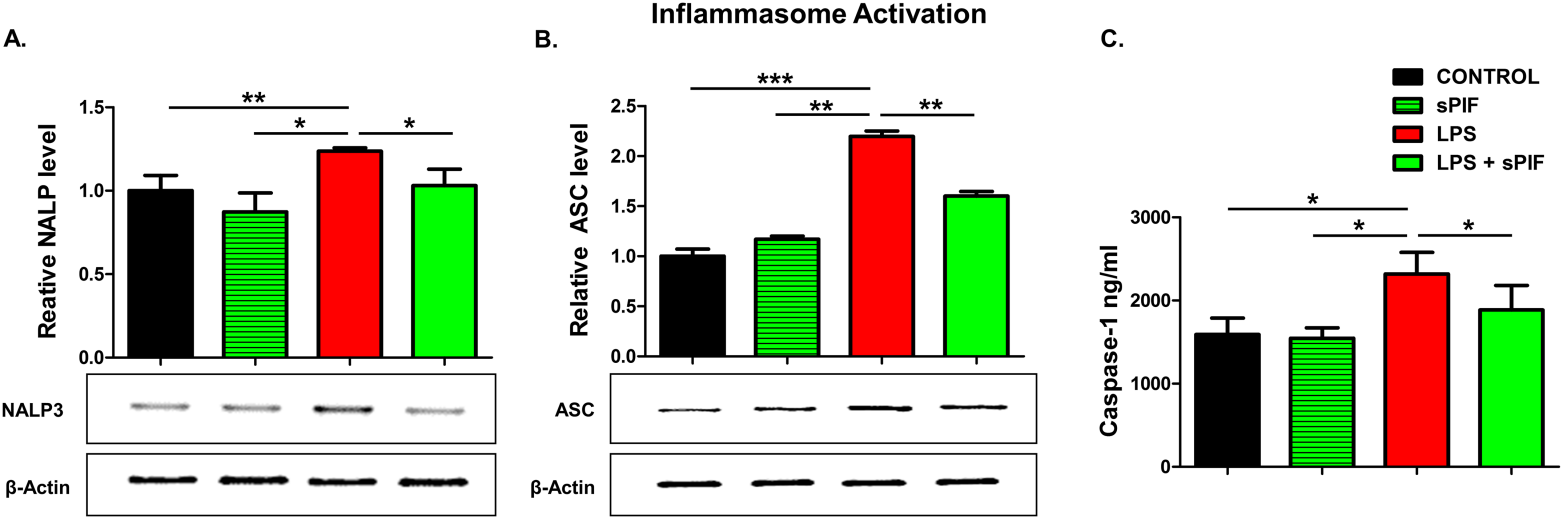
Inflammasome pathway analysis (18–20 placental lysates for each group). Representative gel images of **(A)** relative NALP-3 and **(B)** relative ASC protein levels in placental tissue lysates. The downstream cytokine caspase-1 levels in lysates were measured using enzyme-linked immunoassay **(C)**. Data are mean ± SD. β-Actin was the control. *p<0.05. sPIF: synthetic PreImplantation Factor; LPS: Lipopolysaccharides.

Given that synthetic PIF can reduce major inflammasome signaling cytokines such as IL-1β and IL-18 [35], we decided to test key components of this pathway in the placentae next. Notably, IL-18 contributes to preterm birth in part by modulating TLR4 signaling [36] and TLR4 activation is necessary for PIF effects [27, 28]. We focused on NALP-3 and ASC proteins expression in the placenta as these are the key components of inflammasome signaling [4, 5]. Notably, this multi-protein complex enables caspase-1-mediated production of pro-inflammatory cytokines such as IL-1β and IL-18. We detected increased placental expression of both NALP-3 and ASC proteins in LPS-treated mice (Fig 3A and 3B compare red to black bars). In line with our hypothesis, synthetic PIF reduced this activation significantly (Fig 3A and 3B compare green to black and red bars). We tested downstream inflammasome activation (caspase-1) as well. Again, we detected increased levels of caspase-1 in the placenta after LPS exposure and the addition of PIF reduced this protein’s levels significantly (Fig 3C compare green to red and black bars). Together, we document increased endogenous PIF expression after an LPS insult (Fig 2). Importantly, only application of synthetic PIF results in prevention of increase in cytokine levels after the LPS challenge including the inflammasome pathway. We hypothesize that as in case of progesterone supplementation [34], a combined local and systemic inflammatory control is necessary to reduce LPS-induced fetal loss.

## Discussion

Our current findings are in line with the notion of PIF`s trophic and protective action on the embryo, decidua, and trophoblast [12]. Synthetic PIF administration alone decreased fetal loss and improved fetal weight without modulating the inflammasome pathway (Figs 1B, 1D and 3). These favorable effects suggest a beneficial role in optimization of the implantation process. In the current study, we did not detect changes in the number of implanted embryos and plug/pregnancy rate (S1 Table). However, the current experimental setup is not suitable to answer this specific question as this model does not share features with human early recurrent miscarriage as the CBA/J x DBA/2 mouse model (mating of CBA/J females (H2k) with DBA/2J males (H2d)) [37–39]. On the other hand, synthetic PIF increased serum levels of eotaxin independent of the LPS insult (Table 2). Eotaxins regulate extravillous throphoblast function during uterine decidual vessel remodeling [33]. Furthermore, eotaxin has been shown to promote eosinophil adhesion to vascular cell adhesion molecule-1, a major ligand for the integrins [40]. As trophoblast differentiate into an endovascular phenotype a4 integrin is up-regulated [33] and up-regulation of integrins by PIF during throphoblast invasion was reported previously [15, 18]. Thus, current and previous data support the use of synthetic PIF in early recurrent pregnancy loss [20, 23, 41] and studies are in preparation.

Synthetic PIF`s effects on placental/fetal weight ratio after LPS insult are of interest as well (Fig 1E). LPS was reported to reduce uterine blood flow to the fetus and increase uterine resistance without affecting the placental size thereby leading to fibrin deposits and disseminated intravascular coagulation [42]. Thus, growth retardation is expected and was observed in our study (Fig 1D: compare red to black bars). The role of the inflammasome specifically NALP-3 in LPS induced pathology in placental cells has been previously described [35]. Importantly, we report that the LPS induced activation of placental inflammasome can be prevented by synthetic PIF administration (Fig 3). This is supported by the fact that PIF`s effects dependent on TLR4 signaling [27, 28] and TLR4 is the main receptor of inflammasome activation [43]. Furthermore, synthetic PIF targets Kv1.3b channel to reduce K^+^ flux which activates NALP-3 expression [29, 43]. We hypothesize that synthetic PIF`s effects on placental caspase-1 levels protect against apoptosis and compromise critical oxygen and nutrients transfer to the fetus [44].

Furthermore, the placental/fetal weight ratio relevance for pregnancy outcome was addressed by large population studies previously and an increased ratio was noted in premature birth and fetal demise [32, 45, 46]. Various pathologic and inflammatory conditions are associated with abnormal placental/birthweight ratios including chronic hypertension, preeclampsia, and chorioamnionitis. The present study underscores the fact that the placenta (a primitive organ, supplied by maternal circulation) and the fetus (complex, more evolved with higher oxygen and nutrient demands) are coupled entities. Herein, inflammatory insult affects placental function (but not weight/growth) and still results in reduced fetal weight. We hypothesize that the noted improved fetal-placental exchange is partially due to PIF’s role in promoting implantation [20, 23] and/or reducing vascular inflammation [29]. However, it is still not understood how synthetic PIF increases fetal weight despite of LPS insult (Fig 1D). Although hypothetical, the modulation of the H19/insulin-like growth factor 2 (Igf2) imprinted locus is intriguing. H19 is a long non-coding RNA and placental H19 misregulation is associated with the imprinting disorder such fetal overgrowth disorder (Beckwith–Wiedemann syndrome) and intrauterine growth retardation (Silver–Russell syndrome) [47]. Notably, we recently reported the close functional interactions of H19 and microRNA let-7 [48, 49] and let-7 expression is modulated by synthetic PIF [27]. Thus, synthetic PIF`s role on H19 modulation in intrauterine growth restriction models is currently investigated but beyond the scope of this manuscript. Notably, women with unexplained infertility and women with endometriosis show decreased H19 expression in eutopic endometrium suggesting a direct clinical link [50, 51].

Synthetic PIF`s effect on local and peripheral cytokine level are of interest as well (Tables 1 and 2). Altered balance of pro- and anti-inflammatory cytokines forms the basis of multiple pregnancy disorders and results in fetal and maternal pathological changes [12, 52–56]. For example, IL-18 is significantly elevated at the onset of labor [57] and contributes to preterm birth [36, 57]. Preterm birth or placental hypoxia is associated with high concentrations of granulocyte macrophage-colony stimulating factor (GM-CSF) [58, 59]. GRO a well-defined neutrophil-attractive chemokine increases in case of chorioamnionitis [60]. Besides maternal morbidities, prenatal LPS insult results in sustained inflammation in fetus and newborn, which is associated with an increased risk for adverse outcomes such as brain damage and pulmonary or intestinal complications [61–64]. Given that synthetic PIF prevented the increase of those cytokines/chemokines in the serum (Table 2) and partially in the placenta (Table 1), we envision synthetic PIF treatment in pregnancies at risk as in case of progesterone [52]. This is supported by the fact that synthetic PIF reduces the inflammasome response (Fig 3) and NALP-3 induced IL-18 and IL-1β impact the pathogenesis of preeclampsia, preterm birth, and perinatal brain injury [3, 65, 66].

The simple notion of TH_2_ overbalance during pregnancy is not specific enough. More recent insights into immunological operative mechanisms in pregnancy favor the TH_1_/TH_2_ cooperation [67]. The dual role of IL-4 is an examples [68]. Synthetic PIF prevented the LPS induced Th_2_-type cytokine IL-4 in the serum (Table 2). Notably, IL-4–induced natural killer cells produce higher levels of IFN-γ, IL-10, and GM-CSF while exhibiting high cytotoxicity [68]. Not surprisingly, synthetic PIF prevented the increase of those cytokines in the serum (Table 2) in line with natural killer cells being PIF`s target [23]. Finally, synthetic PIF modulated IL-22, a member of IL-10 family (Table 2) and increased IL-22 levels are an inflammatory marker of placental dysfunction and early pregnancy loss [56, 69].

In conclusion, we provide evidence supporting synthetic PIF as a targeted therapy in inflammatory induced pregnancy loss. This study is in line with PIF`s efficacy previously observed in several preclinical models of immune disorders [24–30]. Additionally, this is the first description of placental expression of PIF after LPS-induced inflammatory insult. Synthetic PIF is currently in first-in-human Phase Ib clinical trial (NCT02239562) for adult patients with autoimmune disease. Upon completion the use of synthetic PIF as treatment for perinatal brain injuries is planned [27, 28]. Synthetic PIF therapy in pregnancy disorders such as preeclampsia, preterm birth, and early pregnancy loss are in preparation as well [12, 19, 41].

## Supporting information

S1 Table Detailed outcomes of the performed experiments. Detailed results of the performed experiments and groups (Control, sPIF, LPS, and LPS+sPIF) are summarized including the number of implanted embryos and plug/pregnancy rate.
(PDF)
