# Supplementary material for: Synthetic PreImplantation Factor (PIF) prevents fetal loss by modulating LPS induced inflammatory response
Source: PLoS One. 2017 Jul 12;12(7):e0180642. doi: 10.1371/journal.pone.0180642 (PMC5507516; doi:10.1371/journal.pone.0180642)
Supplement: S1 Table — Detailed results of the performed experiments and groups (Control, sPIF, LPS, and LPS+sPIF) are summarized including the number of implanted embryos and plug/pregnancy rate. (PDF) [file pone.0180642.s001.pdf]

Fetal Loss

CONTROL		sPIF		LPS		LPS + sPIF	
Mean	SD	Mean	SD	Mean	SD	Mean	SD
19	2.3	7.5	2.6	74	3.8	39	4.6

Fetal weight

CONTROL		sPIF		LPS		LPS + sPIF	
Mean	SD	Mean	SD	Mean	SD	Mean	SD
0.42	0.05	0.552	0.017	0.263	0.01	0.324	0.02

Placental weight

CONTROL		sPIF		LPS		LPS + sPIF	
Mean	SD	Mean	SD	Mean	SD	Mean	SD
0.15	0.037	0.14	0.034	0.151	0.011	0.169	0.04

Ratio Placental/Fetal weight

CONTROL		sPIF		LPS		LPS + sPIF	
Mean	SD	Mean	SD	Mean	SD	Mean	SD
0.3571429	0.04	0.2536232	0.01	0.5741445	0.0499	0.521605	0.031

Pug/Pregnancy Rate

CONTROL	sPIF	LPS	LPS + sPIF
Percent	Percent	Percent	Percent
83.3	88.2	83.3	88.2

Number of Implantations per mouse

CONTROL		sPIF		LPS		LPS + sPIF	
Mean	SD	Mean	SD	Mean	SD	Mean	SD
13	2	15	3	12	3	15	1

Relative NALP level

CONTROL		sPIF		LPS		LPS + sPIF	
Mean	SD	Mean	SD	Mean	SD	Mean	SD
1	0.0919449	0.8731913	0.1141701	1.236641	0.0208218	1.030534	0.0990603

Relative ASC level

CONTROL		sPIF		LPS		LPS + sPIF	
Mean	SD	Mean	SD	Mean	SD	Mean	SD
1	0.072396	1.169483	0.0312596	2.197651	0.053886	1.600503	0.0465064

Caspase-1 ng/ml

CONTROL		sPIF		LPS		LPS + sPIF	
Mean	SD	Mean	SD	Mean	SD	Mean	SD
1590	198	1547	125	2319	260	1885	296
